# A multi-organization epigenetic age prediction based on a channel attention perceptron networks

**DOI:** 10.3389/fgene.2024.1393856

**Published:** 2024-04-24

**Authors:** Jian Zhao, Haixia Li, Jing Qu, Xizeng Zong, Yuchen Liu, Zhejun Kuang, Han Wang

**Affiliations:** ^1^School of Computer Science and Technology, Changchun University, Changchun, China; ^2^ School of Computer Science and Technology, Jilin University, Changchun, China; ^3^ School of Information Science and Technology, Institute of Computational Biology, Northeast Normal University, Changchun, China; ^4^ Clinical Research Centre, Guangzhou First People’s Hospital, School of Medicine, South China University of Technology, Guangzhou, Guangdong, China; ^5^ School of Computer Science and Engineering, Changchun University of Technology, Changchun, China; ^6^ Department of Medicine, Boston University School of Medicine, Boston, MA, United States

**Keywords:** DNA methylation, epigenetic clock, deep learning, attention mechanism, age prediction

## Abstract

DNA methylation indicates the individual’s aging, so-called Epigenetic clocks, which will improve the research and diagnosis of aging diseases by investigating the correlation between methylation loci and human aging. Although this discovery has inspired many researchers to develop traditional computational methods to quantify the correlation and predict the chronological age, the performance bottleneck delayed access to the practical application. Since artificial intelligence technology brought great opportunities in research, we proposed a perceptron model integrating a channel attention mechanism named PerSEClock. The model was trained on 24,516 CpG loci that can utilize the samples from all types of methylation identification platforms and tested on 15 independent datasets against seven methylation-based age prediction methods. PerSEClock demonstrated the ability to assign varying weights to different CpG loci. This feature allows the model to enhance the weight of age-related loci while reducing the weight of irrelevant loci. The method is free to use for academics at www.dnamclock.com/#/original.

## 1 Introduction

Methylation occurs mainly at the 5th carbon atom of cytosine in CpG dinucleotides, and changes in methylation are directly correlated with changes in gene expression (Sun et al., 2011). Recently, there has been an increasing interest in the relationship between aging and methylation (Tang et al., 2018; Luo et al., 2023; Sinha et al., 2023). Many literatures have proposed that age increase is displayed by DNA methylation changes (Fraga et al., 2007; Christensen et al., 2009; Horvath et al., 2012). Since DNA methylation levels at specific sites could be variable over time, the DNA methylation-based epigenetic clocks could be used to effectively quantify biological aging, which can be widely used in anti-aging applications.

Genome-wide DNA methylation is widely measured using microarray-based technology, including Illumina HumanMethylation27 (27 K), HumanMethylation450 (450 K) and HumanMethylationEPIC (850 K) (Moran et al., 2016). By calculating the beta value of DNA methylation for each specific cytosine locus (Levy et al., 2020), the methylation level of each CpG can be quantified. Most of the emerging age prediction methods have been developed based on the transformed data from these techniques.

Machine learning methods have been relatively well developed in the field (Wang et al., 2023a; Lv et al., 2023). In 2013, Horvath (2013) developed a 353-locus age prediction model using data from 51 different tissues, and the difference between their model predicted age and chronological age was around 3.6 years. Although the error rate was high in some tissues, such as the breast, it is still considered the most accurate pan-tissue epigenetic clock by far (Chen et al., 2019; Fahy et al., 2019; Fitzgerald et al., 2021). Hannum et al. (2013) developed an age prediction model using 71 CpG loci with blood data. It firstly revealed that factors such as sex and weight affect the prediction of methylation age. Most published methods used linear regression (Zou and Hastie, 2005) for age prediction (Lin et al., 2016; Shireby et al., 2020; Zhang et al., 2019). They collected loci with a high impact on predicting age to form an epigenetic clock for methylation age prediction. The linear regression method is computationally simple and can predict age using fewer loci, but the method ignores the effect of the remaining loci on the predicted age. The linear regression method also has some limitations in predicting age at methylation, which can lead to high prediction errors.

During the past few years, deep learning was introduced to address this challenge. Compared to machine learning methods, deep learning technology is emerging as a promising approach to improving this area since it is more inclusive for multi-feature tasks to achieve higher accuracy (Wang et al., 2023b; Ispano et al., 2023; Qi and Zou, 2023; Sreeraman et al., 2023). Thong et al. (2021) demonstrated an artificial neural network model which model using three genes that outperformed linear regression models. Levy et al. (2020) used the MethylNet deep learning model to predict the age of DNA methylation and demonstrated its significant advantage over machine learning models. Li et al. (2021) used correlated pre-filtered neural networks (CPFNN) for age prediction and found that appropriately weighting features highly correlated with prediction results is a critical factor in improving prediction accuracy. de Lima Camillo et al. (2022) propose a model AltumAge using deep neural networks by referring to DeepMAge, a model trained on blood samples by Galkin et al. (2021). They reduce the prediction error to 2.153 and the correlation between relevant CpG loci in issues discussed in some detail. However, in the experiments, it was found that the deep learning models that have emerged so far have poor generalization ability in independent datasets and poor prediction accuracy for independent samples. So, there is still room for further optimization in the prediction of methylation age using deep learning models.

In this study, we propose a perceptron prediction model based on the channel attention mechanism, which is a nonlinear regression algorithm. This paper uses the 24,516 CpG loci common to all 3 Illumina platforms for age prediction to ensure that all CpG loci are able to participate in the task. The model uses a channel attention module to assign different weights to individual loci so that the model focuses on task-relevant CPG features and reduces the weights of irrelevant CpG features to provide more valid information for the age prediction task. Compared with the simple perceptron model, the inclusion of the channel attention mechanism in our method leads to a greater improvement in the generalization ability and prediction accuracy of the model.

## 2 Materials and methods

### 2.1 Datasets

**FIGURE 1:**
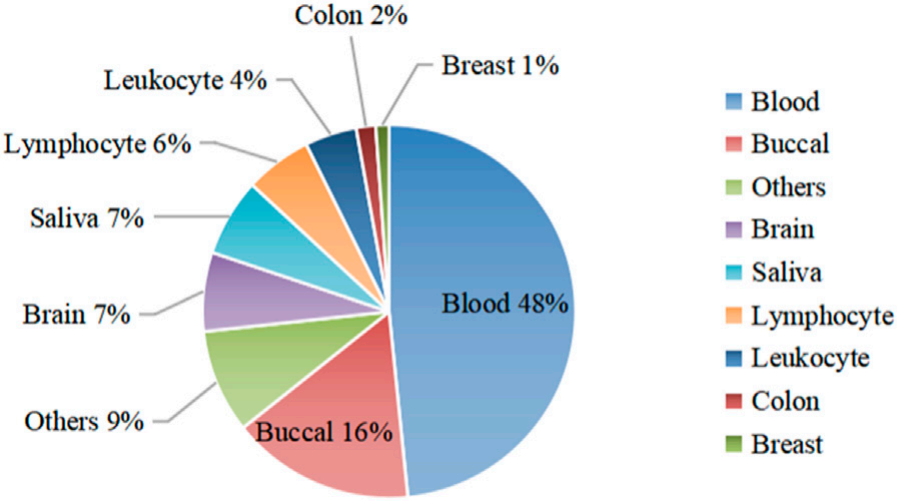
Organizational chart of the data sample. The figure divides the dataset into nine parts according to organization, with blood data and bucca data accounting for a larger share. Tissue data that account for less than one percent are summarized in the “Others” set, for a total of 9%. The remaining 6 tissue data are sorted in order of their share.

We collected 50 datasets from GEO (Barrett et al., 2012) with a total of 13,658 health samples respectively from Infinium 27 K, 450 K, and 850 K platforms (Zhang et al., 2019; Qi and Zou, 2023; Sreeraman et al., 2023), of which 35 datasets were used for model construction and the remaining 15 datasets were used for independent testing. The raw data were separately saved to the clinical data and beta value matrix using the R package GEOquery (Davis and Meltzer, 2007), Then filtered to remove samples in the dataset with more than 50% of the beta value missing. Finally, a method based on simple linear regression (methyLImp) (Di Lena et al., 2019) was used to fill in some of the missing values in the beta value matrix. Figure 1 shows an overview of the 35 data sets by organization.

Different tissues may require different markers to achieve a high level of accuracy in prediction accuracy. Woźniak et al. (2021) developed VISAGE enhanced tool and statistical models based on blood, oral cells and bone. They used three different combinations of loci to construct models that provide accurate DNA methylation age estimates for each tissue separately. The number of CpG loci in 27 K, 450 K, and 850 K data is usually 27,578, 485,577, and 868,564, respectively. Since many datasets are missing CpG loci, this paper takes the 24,516 loci common to the three platforms for model training. The beta value on each CpG locus indicates the degree of DNA methylation. A beta value of 1 indicates that a CpG locus is fully methylated on the allele, while a beta value of 0 indicates that the CpG locus is entirely unmethylated.

### 2.2 Model construction

**FIGURE 2:**
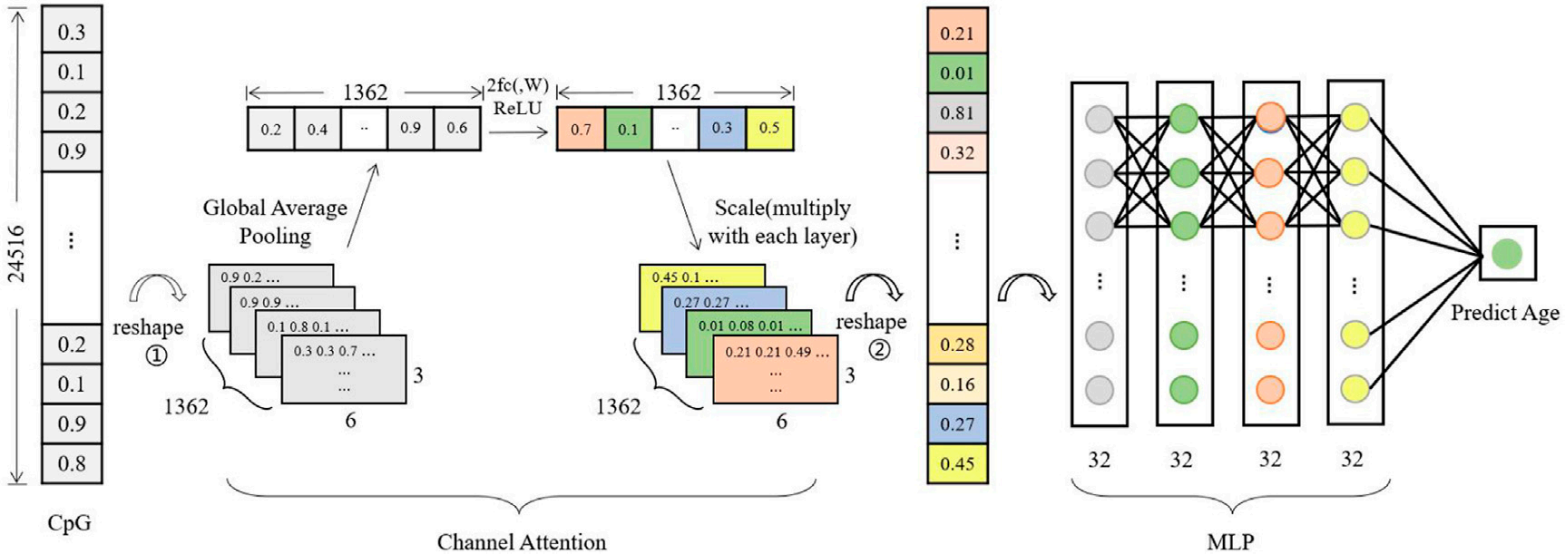
Model architecture. The model has four components, Input, Channel Attention, MLP, and Output. The CpG loci of a sample are input and reshape as 3D data, where the number of channels is 1,362. Global Average Pooling is calculated as a value on each channel, and the data is fitted in a two-layer network to scale with the original 3D data, the process is able to continually enhance the features that are relevant to the prediction results. Afterwards, the 3D data is transformed into one-dimensional data to be fit into a four-layer MLP network to output the predicted age values.

Figure 2 shows the neuro network structure of the model in this paper, which mainly consists of a channel attention module and a perceptron module. The channel attention module assigns different weights to the data according to the importance of the CPG loci. In contrast, the perceptron module uses a 4-layer network for continuous fitting to accomplish the purpose of age prediction.

The first reshape operation in the attention module is to transform a column of data into a block structure, and 1,362 denotes the number of channels, each containing 3 × 6 beta values. The one-dimensional data with 24,516 components is transformed into three-dimensional data with the structure 3 × 6 × 1,362 by placing the beta values in the input data in different channels in order. Global Average Pooling denotes the calculation of the average of 18 beta values in each channel, and each channel is calculated to obtain one value for a total of 1,362 values. 2fc (,W) denotes two fully connected. The 1,362 data are updated according to these two fully connected layers. The updated data are subjected to Scale operation, and the updated 1,362 values are assigned to the first reshaped post-block structure, explicitly using the values of each channel multiplied by 18 beta values of different channels respectively. This will result in a feature value that is weighted by the channel attention module. The perceptron module feeds the processed eigenvalues into a four-layer fully connected layer for regression operations and finally outputs a methylated age prediction.

For the DNA methylation age prediction task, an exact age value was required, so a regression model was used to construct the network. Initially, the model consisted of only a perceptron network, which was erected by 5 fully connected layers with a simple model structure. However, it was found in the experiments that using a simple perceptron model was not sensitive enough to epigenetic factors (CpG loci), and the model training was more likely to be overfitted. Since the number of feature loci used makes network learning more difficult, attention mechanisms are introduced in the deep learning model, specifically, combining the perceptron model (MLP) with the channel attention mechanism SENet. The channel attention module makes the model pay more attention to those CpG loci with high correlation with age, automatically adjusts the weights according to the loss values of model training, and then assigns the weights to the initial features, thus speeding up the model fitting.

### 2.3 Model training

Firstly, the beta values of CpG loci of the training samples are loaded into the model in batches. The input data in Figure 2 takes one sample data as an example, and transforms (reshape) the one-dimensional data with 24,516 components into the three-dimensional data with the structure 3*6*1,362 (adjacent CpG loci put into different channels). After the global average pooling operation, 1*1*1,362 values are calculated to obtain the initial weights of each channel. Then 1*1*1,362 are fed into the two fully connected layers, and the weights are updated according to the loss values of the model training. The updated 1,362 weights are assigned to the corresponding channels of the original 3D data. Finally, the data are transformed (reshape) into one-dimensional data and sent to the four-layer perceptron model for regression operations. The training is ended when the loss values of the model training tend to be stable, and then the validation set and test set are tested and the trained model parameters are saved.

The network model in this paper consists of an input layer, channel attention module, 4 hidden layers and 1 output layer. Each hidden layer consists of 32 neurons, LeakyReLU activation function and BatchNorm1d (32) batch normalization, Dropout (0.1) regularization, and finally the network is optimized using Adam’s algorithm. The model training process is monitored using the loss function of MSELoss. The decreasing trend of the validation loss is observed at the output training loss, and the training is ended at the lowest validation loss value. The final model training parameters were adjusted as follows: learning_rate = 0.05, batch_size = 512, num_epochs (training times) = 150.

The model updates the weights based on the loss function. By weighting the CPG loci with different levels of importance, the important features on different channels are strengthened and the invalid features are weakened to enhance the feature representation of the feature map. Using the changed feature data for model age prediction reduces the number of model parameters and computing pressure to a certain extent, thus enabling the model to perform age prediction more effectively and improve model accuracy.

### 2.4 Experimental platform

Our model is built based on Python 3.7.11. We use Pytorch and the Sklearn library to build the network for data preprocessing.

The AltumAge method is based on Python 3.8, and the network is built using the TensorFlow framework for testing. Several other ways apply R language to reproduce the code. Among them, the Horvath method uses data normalization operation when predicting the data, the code prediction time is longer and the effect is not much different from that without normalization operation, so the data is not normalized in the comparison experiment.

## 3 Results and discussions

### 3.1 Data sample arrangements

We here preprocess the data before loading it into the model. First, the order of the read-in data is disordered, and then the disordered data are divided into training set, validation set, and test set with the number of data samples of 8,577, 953, and 1,059, respectively. Finally, the data are processed into tensor format and put into the model for training. Since the beta value (0–1) in the beta value matrix represents its degree of methylation, which has a special meaning in DNA methylation age prediction, the data in this paper were not normalized.

### 3.2 Evaluation indicators

In order to evaluate the accuracy of the model in predicting age, four evaluation metrics, correlation coefficient (R-squared), mean absolute error (MAE), mean squared error (MSE), and median absolute error (Med) were used. The definition of R-squared is shown in Eq. 1:

$$R^{2}=1-SSE/SST$$ (1)

Where SSE is the error sum of squares and SST is the total sum of squares.

The MAE and MSE are defined as shown in Eqs 2, 3:

$$MAE=\frac{1}{m}\sum_{i=1}^{m}\left(preAge_{i}-chrAge_{i}\right)$$ (2)

$$MSE=\frac{1}{m}\sum_{i=1}^{m}{\left(preAge_{i}-chrAge_{i}\right)}^{2}$$ (3)

Where m denotes the number of predicted samples, preAgei denotes the predicted age of a single sample, and chrAgei denotes the actual age of a single sample. The definition of Med is shown in Eq. 4:

$$Med=median\left(\left|preAge-chrAge\right|\right)$$ (4)

Where preAge indicates the predicted age of all test data, chrAge indicates the actual age of all test data, and median function indicates taking the median of a set of numbers.

The evaluation metrics of the model in this paper on the training set, validation set and test set are shown in Table 1, where the R-squared is only used to show the correlation between the actual age and the predicted age, and the other three metrics are used to evaluate the accuracy of the model in predicting the age of DNAm. All_data indicates all the data used for development, and Train_data, Val_data, Test_data represent the data for training, validation and testing, respectively. Combining the evaluation metrics commonly used in other literature, Med is finally used as the evaluation metric to measure the accuracy in this paper.

**TABLE 1:** Metrics results from model training, validation, and testing.

	All_data	Train_data	Val_data	Test_data
R^2^	0.95	0.95	0.94	0.93
MAE	2.28	2.08	3.13	3.10
MSE	10.35	7.92	21.89	19.61
Med	1.65	1.57	2.11	2.04

### 3.3 Training and testing performance

**FIGURE 3:**
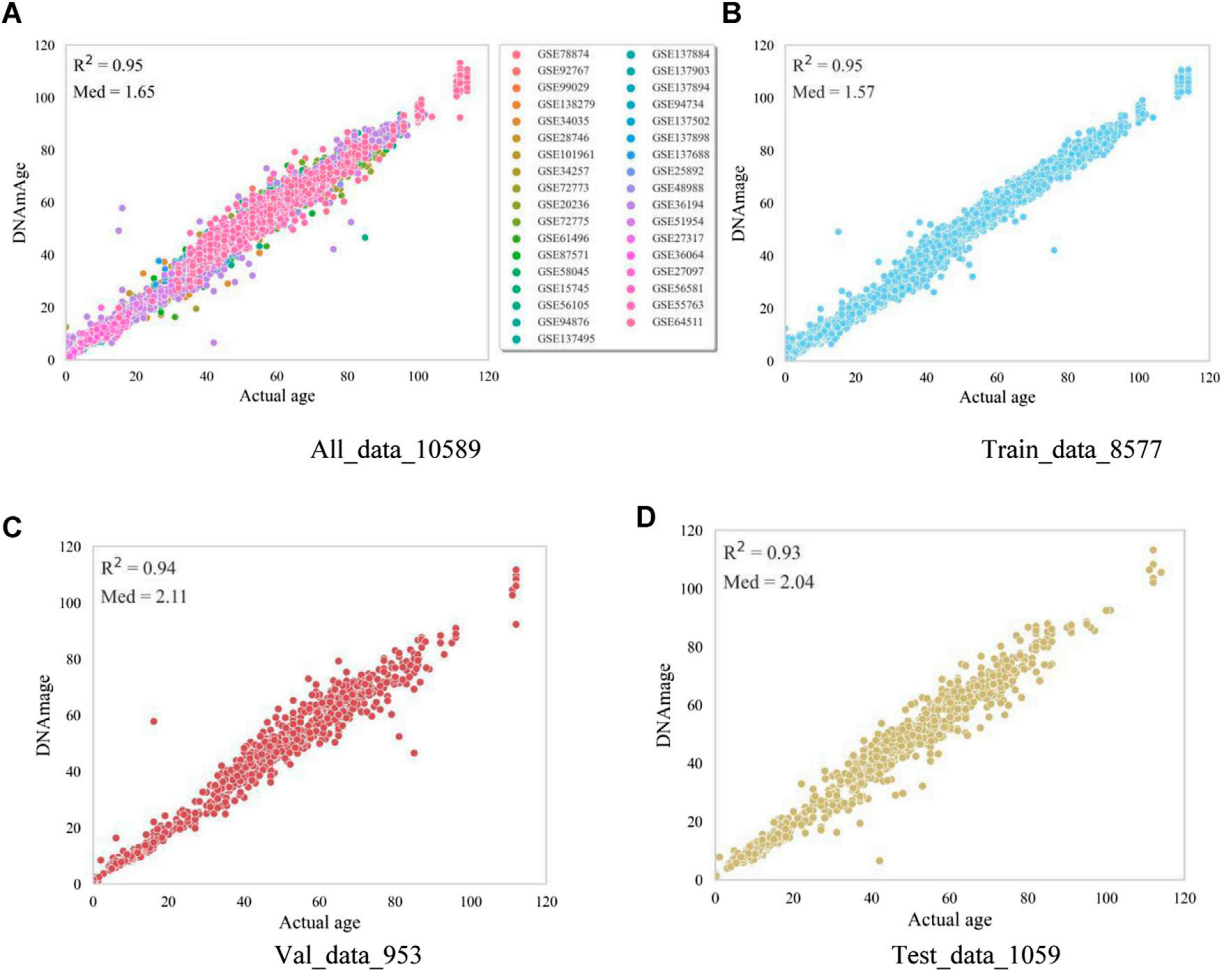
Model visualisation metric results. The horizontal coordinates in the figure represent the actual age of the samples and the vertical coordinates represent the model-predicted age. **(A)** represents the prediction results of all samples, and different colors represent the sample data in different datasets. **(B)** represents the prediction results of the samples trained by the model. **(C)** represents the prediction results of the samples for model validation. **(D)** represents the prediction results of the samples for model testing.

In order to verify the prediction accuracy of the model, we first tested the data used for model training. The test results are shown in Figure 3A shows the prediction results of all samples including the training set, validation set and test set (age correlation = 0.95, median absolute error = 1.65). The horizontal axis is the actual age of the samples, and the vertical axis represents the predicted age of the model. Samples of different data sets are distinguished according to different colors, which shows that the model of this paper performs well on most of the data sets, and only the prediction results of some samples have significant errors. The Figures 3B–D plots represent the prediction results of the training, validation, and testing data in this model, respectively. The actual age and the predicted age all show a high age correlation in the plots, the Med in the training set is 1.57, and the Med in the test set is 2.04. Overall, the model’s prediction accuracy in this paper is high, and the prediction effect is relatively stable in each data set.

### 3.4 Performance comparison with peer methods

To verify the prediction accuracy and generalization ability of the model, 15 separate datasets with a total of 3,069 healthy samples are used in this paper, and the model of this paper is tested separately with other methods on these datasets. The datasets were divided into two batches for separate comparisons. Seven datasets in Table 2 contain the training sets of certain other methods, which are indicated by * in the table; Eight datasets in Table 3 are independent test sets of these methods and are not in the training sets of several methods.

**TABLE 2:** Comparison of the Med of the seven methods on the independent data sets (training set containing the other methods).

Dataset	Num	Ours	Horvath	AltumAge	BNN	Elastic net	LinAge	PhenoAge	CorticalPred
GSE19711	274	5.01	4.23	2.45^a^	**4.15**	5.69	14.4	15.1	6
GSE53740	193	6.84	7.61	2.51^a^	**5.36**	4.71^a^	6.66	12.7	19.8
GSE42861	335	3.83	**3.73**	4.03^a^	13	9.41	3.63^a^	5.59	4.27
GSE43414	141	**4.8**	16.14	18.6	10	16.5	35.3	68.7	3.92^a^
GSE59685	141	**4.48**	12.33	20.71	9.88	16.1	31.3	69.1	3.12^a^
GSE80970	138	**4.26**	13.67	17.00	10.3	18.6	33.2	70.9	2.16^a^
GSE38873	51	**5.72**	4^a^	1.83^a^	11.8	16.4	20	56.6	23

^a^ Indicates the presence of this dataset in the training set of the method.

The bolded font is the best predicted result in addition to the set training set results.

**TABLE 3:** Comparing the Med of the 7 methods in the independent dataset.

Dataset	Num	Ours	Horvath	AltumAge	BNN	Elastic net	LinAge	PhenoAge	CorticalPred
GSE64495	106	**1.73**	2.79	1.73	10.0	15.6	6.01	7.54	4.65
GSE50759	48	**2.18**	3.85	13.38	9.81	38.9	25	11.9	35.9
GSE111223	131	**2.44**	10.05	7.56	6.16	5.29	19.9	7.91	11.2
GSE61431	46	6.45	11.24	11.18	8.92	14.7	30.7	58.3	**6.03**
GSE80261	104	**1.63**	3.01	3.81	12.3	38.4	24	17.1	32.1
GSE74193	450	5.85	**4.19**	5.80	13.7	29.6	13.2	45.5	11.3
GSE112987	64	**1.86**	3.13	11.53	10.2	33.3	8.76	12.4	8.55
GSE152026	928	**3.52**	6.24	10.04	^a^	30.6	3.96	5.37	33.4

^a^ Indicates that the method cannot be measured due to the lack of CpG sites.

Bolded font is the best performing result among the eight methods.

Horvath method, LinAge method, and PhenoAge method (Levine et al., 2018) use traditional elastic net method for age prediction by combination of different CpG sites. Zhang et al. (2019) used elastic net model to screen 514 CpG sites for prediction in order to improve prediction accuracy. The Cortical Pred method is Shireby et al. (2020) developed a methylation age prediction clock for brain tissue, which performed well in other tissues, so it was compared with this paper’s model. AltumAge and BNN methods are methods that use neural network prediction. In this paper, comparison experiments are conducted with the above seven methods. Since the experimental code is not given in DeepMAge by Galkin et al. (2021) and the 71CpG clock of Hannum et al. (2013) is only developed for the 450 K blood dataset, it cannot be tested for datasets with missing loci. Therefore, it was not compared with these two methods.

The comparison results with the seven different prediction methods are shown in Tables 2, 3. Column one in the table is the name of the dataset, columns two and three indicate the number of samples and sample organization of the dataset, and the last eight columns are the Med values of each method on the test dataset. The bolded font in Table 2 is the best predicted result in addition to the set training set results, and the bolded font in Table 3 is the best performing result among the eight methods. From the tested Med results, the model in this paper outperformed other models in 10 datasets, and although it performed slightly worse in three datasets, GSE19711, GSE42861, and GSE61431, it was not much different from the best model in terms of Med results. Due to the small number of samples of brain tissues in the training dataset of the model in this paper, the Med tested in the dataset of brain tissues is slightly larger. To ensure that the test data has a small effect on the test results, an 850 K data was used for testing, and the test results are shown in the row of GSE152026 in Table 3. The BNN method cannot be predicted due to the missing CpG sites, so the # sign is used. Overall, the model in this paper has a low Med and a relatively stable prediction ability tested in each dataset, while confirming this in the 850 K dataset (Hannon et al., 2021).

### 3.5 Differences among multiple organizations

**FIGURE 4:**
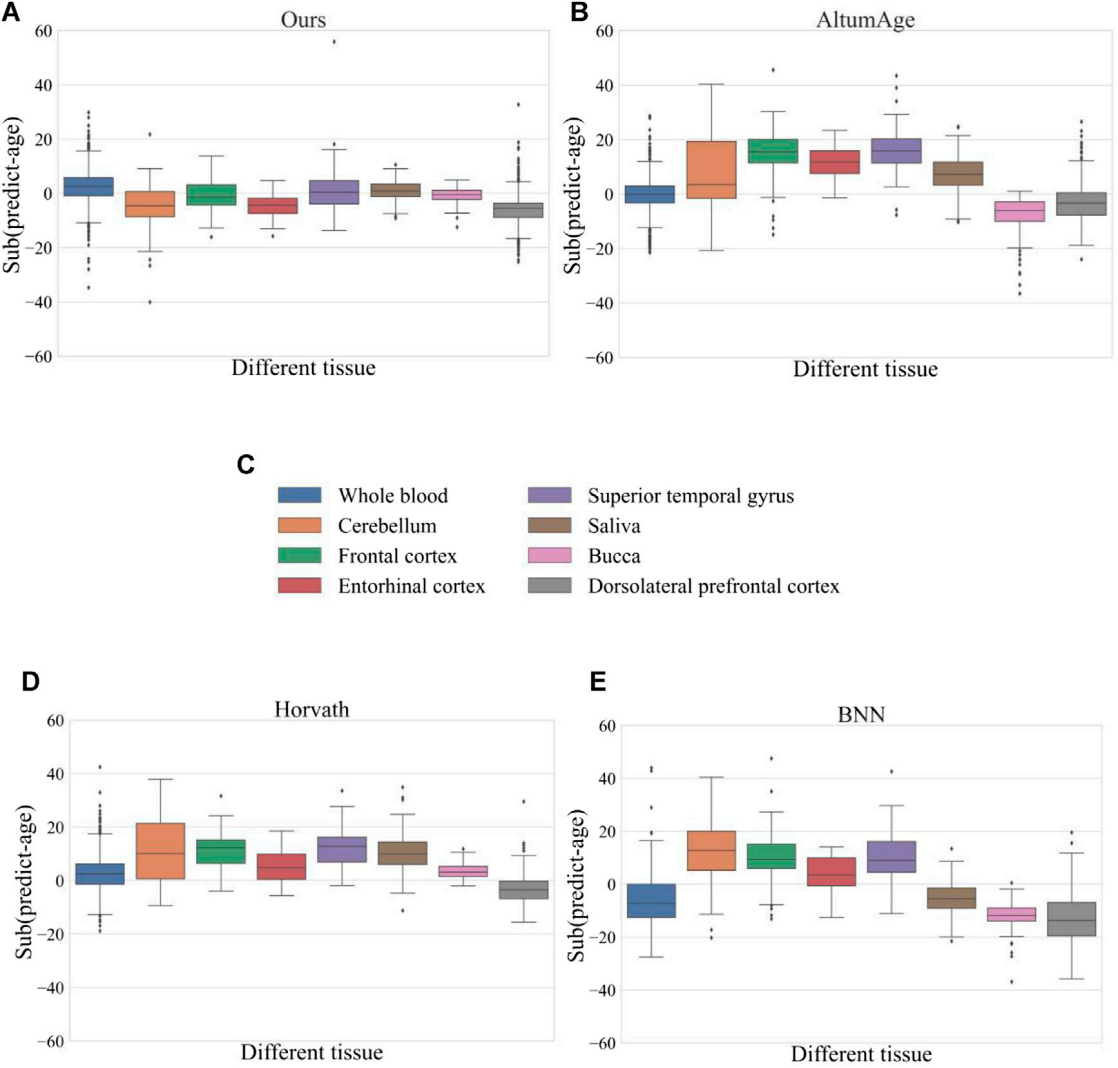
Prediction errors of different methods in eight tissues. Where (A) represents our method (B) represents the AltumAge method (C) represents the eight different organizations (D) represents the Horvath method, and (E) represents the BNN method. There are eight different colored boxes in each figure representing data from different tissues. We calculated the mean error between the predicted age and the actual age for the different organizations. The closer the mean error is to the 0-axis, the better the prediction; the further it is from the 0-axis, the worse the prediction.

To test the prediction accuracy of the model in different tissues, the model was compared with four methods, and Figure 4 shows the prediction error of each model in eight tissues respectively. The horizontal coordinates in the figure represent eight different tissues, and the vertical coordinates indicate the error between the predicted age and the actual age, which is calculated by subtracting the actual age from the predicted age. The comparison results of our model in different tissues showed that the prediction error in entorhinal cortex and dorsolateral prefrontal cortex was slightly larger, but the average error in other tissues was around 0 and the error span was relatively small. Combining the error results of each method, we found that whole blood samples performed relatively stablely in each technique, while entorhinal cortex and dorsolateral prefrontal cortex had more significant errors in each method. This also confirms the significant differences in the epigenomes of different tissue types as suggested by previous researchers (Illingworth et al., 2008; Li et al., 2010).

## 4 Conclusion

Accurate age prediction can help clinicians determine whether the body’s tissues are normal or not. By identifying changes in the genetic characteristics of human tissues, individual disease risk can be effectively reduced. We discuss the usability, accuracy, and the advantages and significance of multi-tissue methylation age prediction methods based on deep learning compared to other methods. Although the prediction has been very accurate using the elastic net method in human aging prediction methods, its exploration of the correlation between CpG loci is not detailed. Deep learning models can not only outperform linear regression models in terms of accuracy, but are also more helpful in investigating the linkages between loci.

In this paper, we propose a perceptron prediction model based on the channel attention mechanism, which has a better learning ability and can improve the accuracy of the model prediction compared with the simple perceptron model. It can be seen from the Med of the test dataset that the model in this paper predicts more accurately than most of the current methods. After experimental validation, this model outperforms other methods on most data sets. Although the error of testing the model in this paper is slightly larger in frontal cortex, it performs well in the test results in various tissues such as blood and saliva. Therefore, compared with other methods, the model in this paper has better predictive power and model generalization ability.

In future works, the changes of CpG loci after adding the attention mechanism and the interconnection between CpG loci need to be further explored. The CpG loci in different tissue samples have different degrees of influence on the prediction results, and comparing them will help to improve the accuracy in different tissues. In the future, the self-attentive mechanism module will be used to update the model parameters, which will make it easier and more explanatory to explore the changes of CpG locus coefficients.

## Data Availability

Publicly available datasets were analyzed in this study. This data can be found here: https://www.ncbi.nlm.nih.gov/geo/.
